# ﻿Two new species of *Curvianchoratus* (Monogenoidea, Dactylogyridae) parasitizing *Psectrogasteramazonica* (Characiformes, Curimatidae) and a new record for *Curvianchoratussingularis* in the Tocantins River, Maranhão, Brazil

**DOI:** 10.3897/zookeys.1172.105500

**Published:** 2023-07-25

**Authors:** Carine Almeida Miranda Bezerra, Simone Chinicz Cohen, Yuri Costa de Meneses, Helyab Gabriel Chaves Neres, Diego Carvalho Viana, Marcia Cristina Nascimento Justo

**Affiliations:** 1 Programa de Pós-Graduação em Ciência Animal, Universidade Estadual do Maranhão, Cidade Universitária Paulo VI, Avenida Lourenço Vieira da Silva 1000, 65055-310 São Luís, MA, Brazil Universidade Estadual do Maranhão São Luís Brazil; 2 Laboratório de Helmintos Parasitos de Peixes, Instituto Oswaldo Cruz, FIOCRUZ, Avenida Brasil 4365, 21040-900 Rio de Janeiro, RJ, Brazil Instituto Oswaldo Cruz Rio de Janeiro Brazil; 3 Núcleo de Estudos Morfofisiológicos Avançados, Universidade Estadual da Região Tocantina do Maranhão, Campus Imperatriz, Rua Godofredo Viana 1300, 65901-480 Imperatriz, MA, Brazil Universidade Estadual da Região Tocantina do Maranhão Imperatriz Brazil

**Keywords:** *Curvianchoratusdominguesi* sp. nov., *Curvianchoratuspsectrogasteri* sp. nov., ectoparasites, fish parasite, helminth fauna, Neotropical Region, taxonomy, Tocantins-Araguaia Basin

## Abstract

Several studies have demonstrated parasitism by monogenoids in characiform fish in the Neotropics. During studies on the helminth fauna of curimatids from the Tocantins River, specimens of *Psectrogasteramazonica* Eigenmann & Eigenmann, 1889 were examined and species of *Curvianchoratus* Hanek, Molnar & Fernando, 1974 were found. Species of the genus are characterized mainly by the complex shape of haptoral anchors with a modified dorsal anchor, composed by two subunits, dorsal-median and dorsal. To date, two species of *Curvianchoratus* are known to parasitize curimatid fishes: the type species *Curvianchoratushexacleidus* Hanek, Molnar & Fernando, 1974 and *Curvianchoratussingularis* (Suriano, 1980). During examination of specimens of *P.amazonica* collected in the Tocantins River, Embiral, Imperatriz, Maranhão State, Brazil, two new species of *Curvianchoratus* were found and are described herein. *Curvianchoratuspsectrogasteri***sp. nov.** and *Curvianchoratusdominguesi***sp. nov.** are characterized by possessing the male copulatory organ formed by a long cirrus and a claw-shaped accessory piece, connected to the base of the male copulatory organ by a ligament. The new species differs from the two known congeneric species mainly by the morphology of the dorsal-median and dorsal subunits of the dorsal anchor. *Curvianchoratuspsectrogasteri***sp. nov.** also differs from other species of the genus by the absence of the ventral bar and *Curvianchoratusdominguesi***sp. nov.** by the size and shape of the ventral bar. An amendment to the diagnosis of *Curvianchoratus* is provided to accommodate the new species. The present study increases the number of *Curvianchoratus* species to four and extends the occurrence of the genus to the Tocantins-Araguaia Basin.

## ﻿Introduction

Characiformes is one of the main orders of Ostariophysi, which occur exclusively in freshwater, with the greatest diversity and species richness concentrated in the Neotropical Region. These fish represent the historical connection between Africa and South America, which can be proven through their distribution and the presence of fossil records (Malabarba and Malabarba 2010, 2014). Among the families of the order, Curimatidae comprises eight extant genera and more than 110 species, living in diverse freshwater habitats from Costa Rica to Argentina (Melo et al. 2018).

*Psectrogaster* Eigenmann & Eigenmann, 1889 is a morphologically diverse lineage of Curimatidae, distributed in streams, rivers, and still waters throughout the drainage basins of the Orinoco River, Essequibo River, Amazon River, Paraguay-Parana River, and some of the rivers of northeastern Brazil (Vari 1989). *Psectrogasteramazonica* Eigenmann & Eigenmann, 1889, popularly known in Brazil as “branquinha” (Santos et al. 1984) is a freshwater fish that lives in tropical waters in the Tocantins and Amazon River basins (Vari and Röpke 2013; Froese and Pauly 2022). This species presents benthopelagic behavior, with the formation of large shoals, and has a maximum length of 19 cm, being one of the largest species of the genus (Vari 1989). The species is one of the most common and abundant along the Tocantins River in midwest, north and northeast Brazil and is the basis of an intensive fishery in this area. Individuals belonging to this species primarily feed on organic detritus, benthic organisms, and periphyton (Santos et al. 1984).

Several studies have demonstrated parasitism by monogenoids in Curimatidae fish in the Neotropics (Table 1). However, research involving the helminth fauna of *P.amazonica* is still incipient, with only two monogenoids described for this host so far (Freitas et al. 2021). Other studies regarding this fish species refer to larval development, the distribution of larvae in a natural breeding area, hematological parameters, and branchial histopathological alterations (Nascimento and Araújo-Lima 1993; Carvalho et al. 2009; Ponte et al. 2017; Bezerra et al. 2020; Pereira et al. 2020).

**Table 1.** Monogenoideans parasites of Curimatidae from South America.

Parasite	Host	Locality	References
*Anacanthoroidesmizellei* Kritsky &Thatcher, 1976	*Steindachnerinainsculpta*	Brazil	Acosta et al. (2013)
*Annulotrematoidesamazonicus* Kritsky & Boeger, 1995	*Psectrogasterrutiloides*	Brazil	Kritsky and Boeger (1995)
*Annulotrematoidesbonaerensis* Rossim & Timi, 2016	*Cyphocharaxvoga*	Argentina	Rossin and Timi (2016)
*Cacatuocotyleparanaensis* Boeger, Domingues & Kritsky, 1997	*Cyphocharaxnagelii*	Brazil	Boeger et al. (1997), Vieira et al. (2013), Dias et al. (2017)
*Characitheciumchascomusensis* (Suriano, 1981) Rossin & Timi, 2014	*Cyphocharaxgilbert*	Argentina	Suriano (1981)
	*C.voga*		Suriano (1997)
*Curvianchoratushexacleidus* Hanek, Molnar & Fernando, 1974	*Cyphocharaxnagelii*	Brazil	Vieira et al. (2013)
*Curvianchoratussingularis* (Suriano, 1980) Suriano 1986	*C.gilbert*	Argentina	Suriano (1980)
	*C.voga*		Rossin and Timi (2016)
	*Cyphocharaxnagelii*	Brazil	Vieira et al. (2013), Dias et al. (2017)
	*Cyphocharaxmodestus*		Dias et al. (2017)
	*Steindachnerinainsculpta*		Dias et al. (2017)
*Diaphorocleiduskabatai* Mendoza-Franco, Reina & Torchin, 2009	*Stendachnerinainsculpta*	Brazil	Acosta et al. (2013)
*Euryhaliotremachaoi* Kritsky & Boeger, 2002	*Steindachnerinainsculpta*	Brazil	Acosta et al. (2013)
*Paranaellaluquei* Kohn, Baptista-Farias & Cohen, 2000	*Stendachnerinabrevipinna*	Brazil	Ceschini et al. (2010)
*Philocorydorasmargolisi* (Molnar, Hanek & Fernando, 1974) Yamada, Brandão, Yamada & Silva, 2015	*Steindachnerinainsculpta*	Brazil	Dias et al. (2017)
*Rhinoxenusguianensis* Domingues & Boeger, 2005	*Curimatacyprinoides*	French Guyana	Domingues and Boeger (2005)
*Urocleidoidescurimatae* Molnár, Hanek & Fernando, 1974	*Steindachnerinaargentea*	Trinidad	Molnár et al. (1974)
*Urocleidoidesparatriangulus* Freitas, Bezerra, Meneses, Justo, Viana & Cohen, 2021	*Psectrogasteramazonica*	Brazil	Freitas et al. (2021)
	*Cyphocharaxgouldingi*		
*Urocleidoidessurianoae* Rossim & Timi, 2016	*Cyphocharaxvoga*	Argentina	Rossin and Timi (2016)
*Urocleidoidestocantinensis* Freitas, Bezerra, Meneses, Justo, Viana & Cohen, 2021	*Psectrogasteramazonica*	Brazil	Freitas et al. (2021)
*Urocleidoidestriangulus* (Suriano, 1981) Rossim & Timi, 2016	*C.nagelii*	Brazil	Vieira et al. (2013), Abdallah et al. (2015), Dias et al. (2017)
	*Cyphocharaxmodestus*		Abdallah et al. (2015), Dias et al. (2017)
	*C.gilbert*		Freitas et al. (2021)
		Argentina	Suriano (1981), Suriano (1997)

Monogenoidea Bychowsky, 1937 are, in general, ectoparasites that mainly infect fish species (Cohen et al. 2021). They have a simple and direct life cycle and, consequently, may present a high reproduction rate in these hosts (Lapera et al. 2017). Monogenoids are among the main components of the parasitic fauna of freshwater fish that occur in the Neotropics, most of them belonging to the family Dactylogyridae Bychowsky, 1933 (Cohen et al. 2013).

*Curvianchoratus* was proposed by Hanek, Molnar and Fernando (1974) to accommodate *Curvianchoratushexacleidus* Hanek, Molnar & Fernando, 1974, a parasite of the characiform *Steindachnerinaargentea* (Gill, 1858) (syn. *Curimatusargenteus* Gill, 1858) from the Arouca River, Trinidad. The main characteristic of *Curvianchoratus* is the haptor with a modified dorsal anchor, composed of two subunits, which is a unique shape within Dactylogyridae.

To date, two species of *Curvianchoratus* are known, both parasites of curimatid fishes: the type species *C.hexacleidus* and *Curvianchoratussingularis* (Suriano, 1980), originally described as *Notodiplocerussingularis* by Suriano (1980) parasitizing *Cyphocharaxgilbert* (Quoy & Gaimard, 1824) [syn. *Pseudocurimatagilbert* (Quoy & Gaimard, 1824)] from Argentina. Suriano (1986) transferred the parasite to *Curvianchoratus*. Rossin and Timi (2016) redescribed this species from *Cyphocharaxvoga* (Hensel, 1870) from Argentina. In Brazil, *C.singularis* has been reported in the rivers of the state of São Paulo parasitizing Curimatidae fishes (Vieira et al. 2013; Dias et al. 2017).

During studies on the helminth fauna of curimatids from the Tocantins River, specimens of *P.amazonica* were examined and species of *Curvianchoratus* were found. The present paper presents an emended diagnosis of *Curvianchoratus*, with the proposal of two new species and a new geographical record for *C.singularis*.

## ﻿Material and method

**Figure 1.**
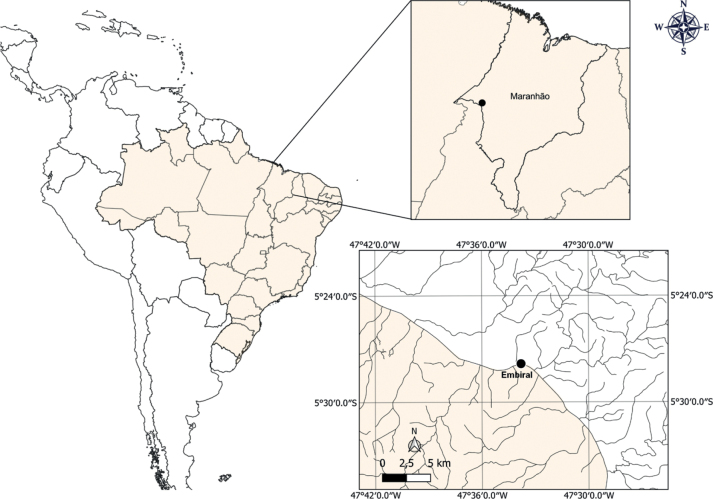
Map of Middle Tocantins River, showing the collection point of the hosts close to the urban perimeter of Imperatriz, in the village of Embiral, state of Maranhão, Brazil.

**Figure 2.**
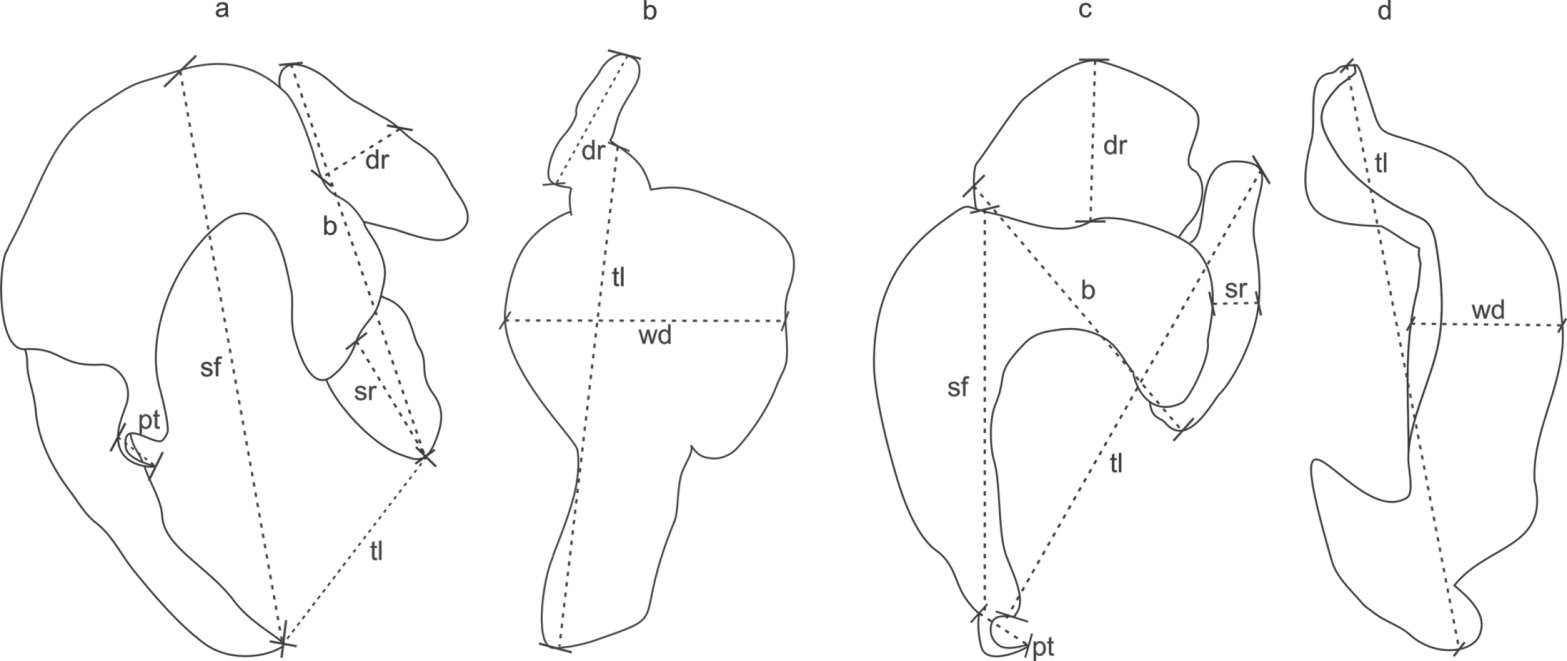
Scheme of measurements of the sclerotized structures of haptoral anchors of *Curvianchoratus* spp. **A** dorsal-median subunit and **B** dorsal subunit of *Curvianchoratuspsectrogasteri* sp. nov. **C** dorsal-median subunit **D** dorsal subunit of *Curvianchoratusdominguesi* sp. nov. (sf) shaft, (pt) point, (dr) deep root, (sr) superficial root, (b) base, (tl) total length, (wd) width.

From July 2018 to April 2022, 136 samples of *P.amazonica* (average 14 cm in standard length and average 62 g in weight) were collected in the Tocantins River, close to the urban perimeter of Imperatriz, in the village of Embiral, state of Maranhão, Brazil (5°27'50’’S, 47°33'48’’W) (Fig. 1). Fish were caught with the aid of local fishermen using gill nets, or a hook and line and were examined asites immediately after capture at the Nucleus of Advanced Morphophysiological Studies at UEMASUL. The gills were removed and placed in vials containing hot water (65 °C) in order to detach the parasites from the gill filaments. Later, absolute ethanol was added to reach a concentration of 70%. Monogenoids were collected from the sediment and gill arches in the laboratory with the aid of a stereoscopic microscope. Specimens were mounted in Hoyer’s medium to study the hard parts, such as the copulatory complex and haptoral sclerites and some were stained with Gomori’s trichrome to study the internal organs of the parasite (Kritsky et al. 1978). Measurements are presented in micrometers; range values are followed by mean and number of structures measured in parentheses. Sclerotized structures, such as bars and anchors were measured according to the scheme shown in Fig. 2. The parasitological indices were calculated as proposed by Bush et al. (1997), followed by the standard deviation. The authorship of the taxa followed the recommendation of Article 50.1 of the International Code of Zoological Nomenclature (**ICZN**), which deals with the identity of the authors. The specimens were observed using an Olympus BX 41 microscope with phase contrast and Zeiss Axioskop 2 Plus microscope with differential interference contrast, both equipped with a camera lucida for drawings. The images were captured with a SONY MPEG Movie EX DSC-S75 digital camera. Type specimens were deposited in the Coleção Helmintológica do Instituto Oswaldo Cruz (**CHIOC**) in Brazil.

## ﻿Results

In the present study, a total of 87 monogenoid specimens were collected, being: 68 *Curvianchoratuspsectrogasteri* sp. nov., eight *Curvianchoratusdominguesi* sp. nov. and 11 *Curvianchoratussingularis*.

### ﻿Taxonomic account

**Class Monogenoidea Bychowsky, 1937**

**Subclass Polyonchoinea Bychowsky, 1937**

**Order Dactylogyridea Bychowsky, 1937**

#### ﻿Dactylogyridae Bychowsky, 1933

##### ﻿ Curvianchoratus

Taxon classificationAnimaliaDactylogyrideaDactylogyridae

Hanek, Molnár & Fernando, 1974

AB8559A8-FE4F-593E-96B5-8E21C12ED6B0

###### Emended diagnosis.

Tegument smooth, thin. Body elongated; divisible into cephalic area, trunk, peduncle, and haptor. Terminal and lateral cephalic lobes developed or not; head organs and cephalic glands present. Eyespots present or absent. Mouth subterminal, midventral, prepharyngeal. Pharynx muscular; esophagus short. Intestinal caeca two, not presenting diverticula. Common genital pore midventral near the level of intestinal bifurcation. Gonads intercaecal, overlapping or not; testis dorsal to germarium. Seminal vesicle large, dilated portion of vas deferens; prostatic reservoir single. Copulatory complex comprising male copulatory organ (MCO) and accessory piece; MCO consisting of sclerotized coiled tube with counterclockwise rings, which make a short turn on itself near the base; cirrus and accessory piece articulated basally. Accessory piece with bifurcated distal portion, pincer-shaped. Ovary pretesticular. Vagina medial or sinistral. Vitellaria well developed, coextensive with intestinal ceca, absent from regions of other reproductive organs. Peduncle short, haptor subquadrate. Ventral bar present or absent. Dorsal bar absent. Ventral anchor of dactylogyrid type. Dorsal anchor modified, composed by two subunits, dorsal-median and dorsal, forming a full circle, the shaft of the dorsal subunit passes over the superficial root of the dorsal-median subunit anchor. Hooks 14; 5 pairs ventral, 2 pairs dorsal, with inflated base and slender shank. Parasites of curimatid fishes.

###### Remarks.

*Curvianchoratus* was proposed to accommodate *Curvianchoratushexacleidus* Hanek, Molnár & Fernando, 1974, a parasite of the characiform *S.argentea* from the Arouca River, Trinidad (Hanek et al. 1974). The main characteristic of *Curvianchoratus* is the haptor with a modified dorsal anchor, composed of two subunits, which is a unique shape for the genus. The absence of bars in one of the species described here, made it necessary to expand the original diagnosis of the genus to include the characteristics of the new species described below, as well as to detail some morphological characters.

##### ﻿ Curvianchoratus psectrogasteri

Taxon classificationAnimaliaDactylogyrideaDactylogyridae

Bezerra, Cohen, Meneses & Justo sp. nov.

50559FE9-0E5C-57B9-B72C-5ACB395906E2

https://zoobank.org/CB6BBC35-0D07-4EC8-A9E6-1BD568F8899B

**Figure 3.**
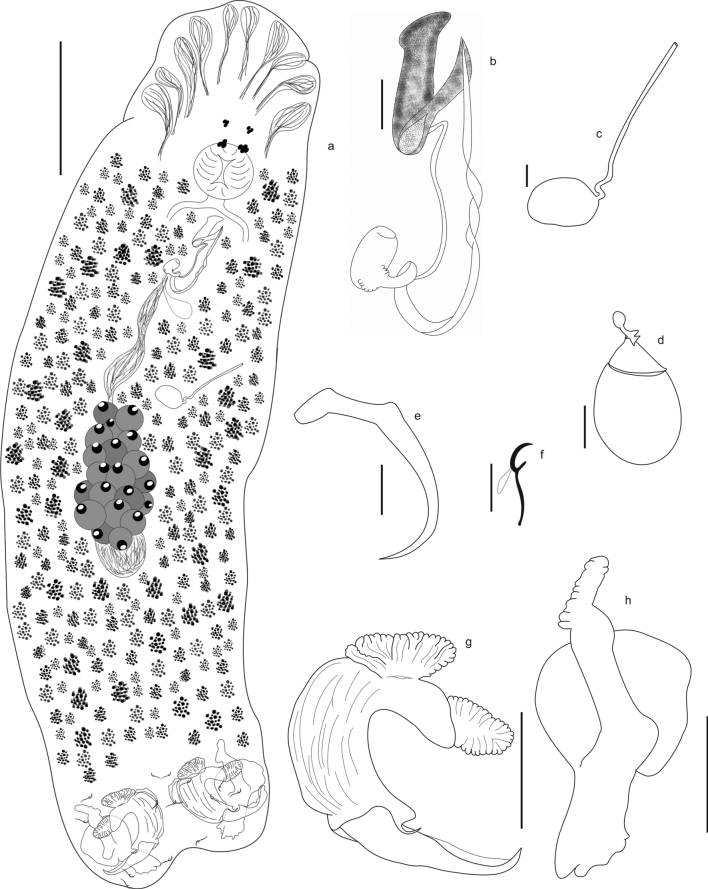
*Curvianchoratuspsectrogasteri* sp. nov. from *Psectrogasteramazonica* from Tocantins River **A** whole specimen, ventral view **B** copulatory complex, ventral view **C** vagina **D** egg **E** ventral anchor **F** hook **G** dorsal-median subunit **H** dorsal subunit. Scale bars: 100 μm (**A**); 10 μm (**B, C, E, F**); 30 μm (**D, G, H**).

Figs 3A–H , 5A, B, E

###### Type host.

*Psectrogasteramazonica* Eigenmann & Eigenmann, 1889 (Curimatidae).

###### Type locality.

Embiral, rural zone (5°27'50"S, 47°33'48"W), municipality of Imperatriz, Maranhão State.

###### Infestation parameters.

22.06% prevalence; 68 total number of parasites; 2.27 ± 1.75 mean intensity; 1-15 range of intensity.

###### Deposited material.

***Holotype***: CHIOC 39977a; ***paratypes***: CHIOC 39977 b-d, 39978 a, b, 39979, 39980, 39981, 39982.

###### Etymology.

The specific name of the species is derived from the generic name of the host.

###### Description.

Based on 43 specimens: 21 mounted in Gomori’s trichrome and 22 mounted in Hoyer’s medium: Body elongated, 425–883 (665; *N* = 30) long, 135–520 (308; *N* = 30) wide. Tegument smooth. Cephalic region with poorly-developed cephalic lobes; five pairs of head organs; cephalic glands lateral to pharynx. Eyes 4; anterior pair smaller than posterior. Pharynx round, muscular, 32–75 (50; *N* = 18) long, 32–87 (50; *N* = 18) wide; esophagus short. Peduncle inconspicuous, followed by a small haptor compared to body size, 125–292 (225; *N* = 21) wide. Ventral bar absent. Ventral anchor with inconspicuous deep root and elongated superficial root, long and slightly curved shaft; straight tip, not passing from the level of the tip of the superficial root, 21–30 (24; *N* = 43) long, base, 10–13 (12; *N* = 43) wide. Dorsal anchor complex heavily modified, composed by 2 subunits: dorsal-median and dorsal. Dorsal-median subunit strongly curved, 52–87 (64; *N* = 34) long and base, 41–63 (49; *N* = 48), with the robust shaft, 63–92 (78; *N* = 34) long, subdivided into two pieces, one anterior, with sclerotized ridges, and one posterior, lightly sclerotized, point sickle-shaped, articulated with the dorsal subunit, 4–7 (6; *N* = 36) long; deep and superficial roots well developed, crested-shaped, 12–27 (16; *N* = 46) long and 10–20 (14; *N* = 37) long, respectively. Dorsal subunit large, irregular, 40–65 (48; *N* = 15) long well-developed deep root 19–22 (20; *N* = 17), superficial root absent, and median region expands to form a rounded structure, 32–57 (40; *N* = 23) wide. Hooks similar in shape, pairs 1 and 5 smaller than others, 11–14 (12; *N* = 27) long, pairs 2–4,6,7 with erected thumb, curved shaft, short point, shank with dilated bulb at distal end, 13–18 (16; *N* = 95). Copulatory complex comprising male copulatory organ (MCO) and articulated accessory piece. Male copulatory organ composed by a tubular sclerotized cirrus that makes a short turn on itself near the base and spirals halfway towards the tip of the cirrus, with sclerotized base possessing delicate membrane, 47–60 (53; *N* = 5) in total length. Accessory piece, 25–30 (26; *N* = 5) long, claw-shaped, with dissimilar tip sizes, the smaller tip serves as guide to MCO, hinged by a ligament to the base of the cirrus. Testis oval, dorsal to germarium; seminal vesicle a distal enlargement of vas deferens; prostatic reservoir single, posterior to MCO. Germarium pretesticular, 62–122 (102; *N* = 7). Vaginal aperture midway between ovary and copulatory complex, sinistro-ventral, with vaginal canal slightly sclerotized, followed by rounded seminal receptacle, anterior to germarium. Oviduct, ootype, uterus not observed. Vitellaria dense, extends over the entire body, except in the region of the reproductive organs and haptor. Eggs operculated, 72–87 (82; *N* = 3) long by 60–70 (64; *N* = 3) wide.

###### Remarks.

*Curvianchoratuspsectrogasteri* sp. nov. is allocated in the genus mainly by the morphology of the copulatory complex, composed by a tubular cirrus and a pincer-shaped accessory piece, connected to the base of the cirrus by a ligament and by the presence of the greatly modified dorsal anchor, composed by two subunits: dorsal-median and dorsal. The new species differs from the two known species by the absence of the ventral bar and by the morphology of the dorsal-median subunit (strongly curved, with the shank and tip visibly separated; a robust shank ornamented with a thorn at the base and crested-shaped deep and superficial roots in the new species vs. a stickle shaped end inserted in the cavity of the shaft in *C.hexacleidus* and *C.singularis*) and by the morphology of the dorsal subunit (elongated, with a broadly circular central region in the new species vs. elongated, with deep root in *C.singularis* and without in *C.hexacleidus*).

##### ﻿ Curvianchoratus dominguesi

Taxon classificationAnimaliaDactylogyrideaDactylogyridae

Bezerra, Cohen, Meneses & Justo sp. nov.

6368EAFF-AC5F-5977-9237-104B5312259F

https://zoobank.org/E6B46BAD-9990-4E75-8781-D4DB18DE70D0

**Figure 4.**
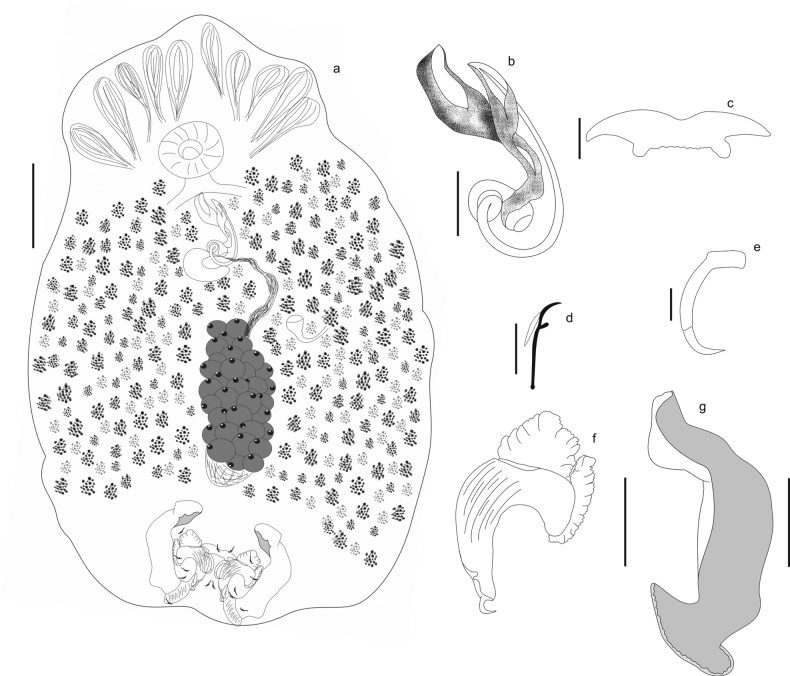
*Curvianchoratusdominguesi* n. sp from *Psectrogasteramazonica* from Tocantins River **A** whole specimen, ventral view **B** copulatory complex, ventral view **C** ventral bar **D** hook **E** ventral anchor **F** dorsal-median subunit **G** dorsal subunit. Scale bars: 100 μm (**A**); 30 μm (**B, F, G**); 10 μm (**C**–**E**).

**Figure 5.**
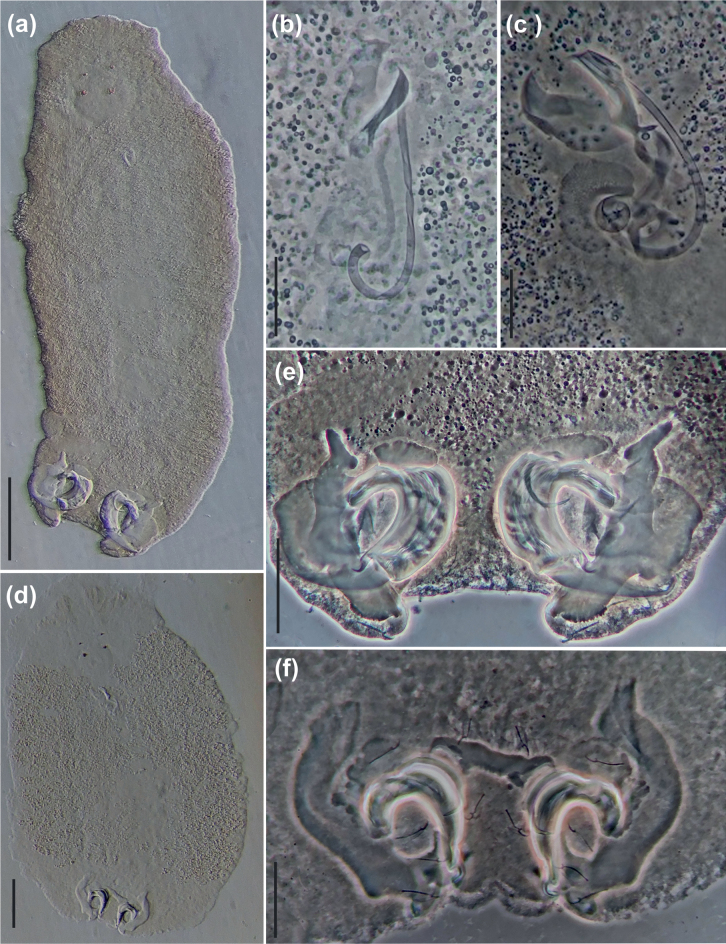
Light photomicrographs of *Curvianchoratus* spp. **A, B, E***Curvianchoratuspsectrogasteri* sp. nov. **A** total, ventral view **B** copulatory complex, ventral view **E** haptor **C, D, F***Curvianchoratusdominguesi* sp. nov. **C** copulatory complex, ventral view **D** total, ventral view **F** haptor. Scale bars: 100 μm (**A**); 20 μm (**B**); 30 μm (**C, F**); 100 μm (**D**); 40 μm (**E**).

Figs 4A–G , 5C, D, F

###### Type host.

*Psectrogasteramazonica* Eigenmann & Eigenmann, 1889 (Curimatidae).

###### Type locality.

Embiral, rural zone (5°27'50"S, 47°33'48"W), municipality of Imperatriz, Maranhão State.

###### Infestation parameters.

5.15% prevalence; 8 total number of parasites; 1.14 ± 0.26 mean intensity; 1–2 range of intensity.

###### Deposited material.

***Holotype***: CHIOC 39984; ***paratypes***: CHIOC 39985, 39986, 39987, 39988, 39989.

###### Etymology.

The specific name is in honor of Dr Marcus Vinicius Domingues for his contributions to the knowledge of neotropical monogenoidean fauna.

###### Description.

Based on 7 specimens: 1 mounted in Gomori’s trichrome and 6 mounted in Hoyer’s medium: Body short, broad, 460–750 (575; *N* = 6) long, 225–500 (362; *N* = 6) wide. Tegument smooth. Cephalic region with 3 pairs of lateral lobes (two lateral and one terminal); five pairs of head organs; cephalic glands lateral to pharynx. Eyespots absent. Pharynx muscular, 50–63 (55; *N* = 3) long, 48–69 (58; *N* = 3) wide, esophagus short. Haptor small, compared to body size length, 400 (*N* = 2) wide. Ventral bar short and robust, anvil-shaped, with slightly jagged edges, 50–58 (52; *N* = 4) long. Ventral anchor with inconspicuous roots, shaft with two subunits, 25–32 (30; *N* = 5) long, with a short base, 11–17 (14; *N* = 5). Dorsal anchor complex heavily modified composed by 2 subunits: dorsal-median and dorsal. Dorsal-median subunit robust curved and heavily sclerotized, 50–66 (58; *N* = 13) long, shaft, 41–47 (44; *N* = 14); point as a robust hooked thorn, 6–10 (8; *N* = 14); superficial root, 5–8 (7; *N* = 3) and deep root, 13–17 (15; *N* = 6), well developed with borders very close to each other; point, 6–10 (8; *N* = 14). Dorsal subunit elongated, 65–100 (77; *N* = 9) long and 18–23 (19; *N* = 9) wide, 30% larger than the dorsal-median subunit, posterior portion fan-shaped. Hooks similar in shape, with erected thumb, curved shaft, and dilated bulb in the end, pair 1 smaller than the others, 11–12 (11; *N* = 4) long; pairs 2–7, 14–17 (16; *N* = 37). Copulatory complex comprising male copulatory organ and articulated accessory piece. MCO tubular, sclerotized, makes a short coil on itself, near the cirrus base, 55–75 (69; *N* = 4) in total length. Accessory piece claw-shaped, bifurcated, with different tip sizes; the smaller serves as a guide for the MCO, 42–70 (56; *N* = 5) long. Testis oval dorsal to the germarium; seminal vesicle formed by a distal enlargement of the vas deferens; prostatic reservoir single, in form of half-moon. Germarium pretesticular; seminal receptacle anterior to germarium; vaginal opening sinistro-ventral; vaginal canal wall sclerotized; oviduct, ootype, uterus, and eggs not observed. Vitellaria dense extends over the entire body, except in the region of the reproductive organs and haptor.

###### Remarks.

*Curvianchoratusdominguesi* sp. nov. is similar to the other species of the genus mainly by the morphology of the dorsal anchor, the copulatory complex, which is composed by a long cirrus and a pincer-shaped accessory piece, connected to the base of the cirrus. The new species is similar to *C.singularis* and *C.hexacleidus* by the presence of the ventral bar. The new species differs from all congeneric species by the shape of the ventral anchor (formed by a shaft with two subunits in the new species vs. well-developed roots in *C.hexacleidus* and moderately developed deep root and well-developed superficial root in *C.singularis* and *C.psectrogasteri* sp. nov.), the morphology of the ventral bar (short and robust, anvil-shaped ventral bar in *C.dominguesi* sp. nov. vs. serpentine-shaped in *C.hexacleidus*, M-shaped in *C.singularis* and absent in *C.psectrogasteri* sp. nov.) and by the ratio of the subunits of the dorsal anchor (dorsal subunit is 30% larger than the dorsal-median subunit in *C.dominguesi* sp. nov. vs. the approximately same size of the subunits in the other three species of the genus).

##### ﻿ Curvianchoratus singularis

Taxon classificationAnimaliaDactylogyrideaDactylogyridae

(Suriano, 1980) Suriano, 1986

1AF13E3C-3293-528E-92F1-083354A759F6

Notodiplocerus singularis Suriano, 1980. Syn.

###### Host.

*Psectrogasteramazonica* Eigenmann & Eigenmann, 1889 (Curimatidae)

###### Site of infection.

Gill.

###### Locality.

Embiral, rural zone (5°27'50"S, 47°33'48"W), municipality of Imperatriz, Maranhão State.

###### Infestation parameters.

29.37% prevalence; 104 total number of parasites; 2,81 (±6,3) mean intensity; 1–38 range of intensity.

###### Type host and locality.

*Cyphocharaxgilbert* (syn. *Pseudocurimatagilbert*) from Chascomus Lagoon, Buenos Aires Province, Argentina (Suriano 1980).

###### Other records.

*Cyphocharaxvoga* from Chascomus Lagoon, Buenos Aires Province, Argentina (Rossin and Timi 2016), *Cyphocharaxmodestus*, *Cyphocharaxnagelii* and *Steindachnerinainsculpta* from Batalha and Peixe Rivers, São Paulo State, Brazil (Vieira et al. 2013; Dias et al. 2017).

###### Specimens deposited.

Voucher CHIOC 39990, 39991, 39992.

###### Remarks.

Suriano (1980) described the species as *Notodiplocerussingularis* and the original drawings are simplified lacking important details of the morphology of these unique anchors that characterize the genus. In the description provided by Rossin and Timi (2016), the morphology of the dorsal anchor complex and copulatory complex was described in detail and illustrated by means of drawings and photographs. The morphology of the present material is in agreement with the material studied by Rossin and Timi (2016).

## ﻿Discussion

Monogenoideans represent one of the main components of the helminth fauna present in freshwater fish in the Neotropics, most of which are dactylogyridean parasites of teleost fish (Boeger and Vianna 2006; Cohen et al. 2013; Braga et al. 2014). The great heterogeneity of hydrographic basins present in Brazil reflects a rich and diversified monogenoid fauna in this region (Tavares-Dias et al. 2022). Despite taxonomic efforts, the diversity of monogenoid parasites of freshwater fishes from South America remains poorly known (Justo and Cohen 2019). According to Melo et al. (2018), most of the information regarding monogenoidean diversity in the Neotropical Region is limited to medium-sized fish species, while the parasitic fauna of small-sized species, especially Characiformes and Siluriformes, is not yet described.

Studies on the parasite diversity of freshwater fish from the Tocantins-Araguaia Basin have gradually increased in recent years [see Fehlauer and Boeger (2005); Boeger et al. (2006); Kritsky et al. (2007); Domingues and Marques (2011); Kritsky et al. (2013); Aguiar et al. (2017); Cárdenas et al. (2019, 2021, 2022); Cohen et al. (2020); Freitas et al. (2021)].

In this sense, the proposal of two new species of *Curvianchoratus* contributes to the knowledge of parasite fauna of the region and enlarges the morphological characters used to describe the unique species that ventral bar is absent (*Curvianchoratuspsectrogasteri* sp. nov.), which is contrary to the known species of the genus. Although the presence of the bar is an important characteristic, we adopted a conservative approach when proposing a new species of this genus, because *C.psectrogasteri* exhibit the complex morphology of the dorsal anchor, characteristic of *Curvianchoratus*.

With the description of the two new species in the present study, *Curvianchoratus* now encompasses four species, all parasitizing curimatid fishes. *Curvianchoratussingularis* is herein reported in *P.amazonica* from the Tocantins River, expanding the occurrence of the species to the Tocantins-Araguaia Basin. The finding of three species of the genus in *P.amazonica* confirms the high specificity of the genus to neotropical curimatid hosts. On the other side, *Psectrogasteramazonica* harbors five monogenoidean species, two from a generalist monogenoidean genus, *Urocleidoides* (parasites from three different fish orders) (Freitas et al. 2021) and three from the specialist genus *Curvianchoratus*.

## Supplementary Material

XML Treatment for Curvianchoratus

XML Treatment for Curvianchoratus psectrogasteri

XML Treatment for Curvianchoratus dominguesi

XML Treatment for Curvianchoratus singularis

## ﻿Additional information

### Author ORCIDs

Carine Almeida Miranda Bezerra https://orcid.org/0000-0003-4842-1595

Simone Chinicz Cohen https://orcid.org/0000-0001-8204-336X

Yuri Costa de Meneses https://orcid.org/0000-0002-8737-1159

Helyab Gabriel Chaves Neres https://orcid.org/0000-0002-4454-0223

Diego Carvalho Viana https://orcid.org/0000-0002-3302-9892

Marcia Cristina Nascimento Justo https://orcid.org/0000-0002-0684-3389

### Data availability

All of the data that support the findings of this study are available in the main text.
